# Cluster-Based Predictive Modeling of User Ratings for Physical Activity Apps Using Mobile App Rating Scale (MARS) Dimensions: Model Development and Validation

**DOI:** 10.2196/70987

**Published:** 2025-11-06

**Authors:** Ayush Bhattacharya, Jose Fernando Florez-Arango

**Affiliations:** 1 Department of Population Health Sciences, Weill Cornell Medicine, 575 Lexington Ave, Room FP 1025, New York, NY, 10022, United States, 1 6469622435

**Keywords:** Mobile App Rating Scale, user rating prediction, machine learning, k means clustering, predictive modelling, MARS

## Abstract

**Background:**

The expansion of mobile health app or apps has created a growing need for structured and predictive tools to evaluate app quality before deployment. The Mobile App Rating Scale (MARS) offers a standardized, expert-driven assessment across 4 key dimensions—engagement, functionality, aesthetics, and information—but its use in forecasting user satisfaction through predictive modeling remains limited.

**Objective:**

This study aimed to investigate how k-means clustering, combined with machine learning models, can predict user ratings for physical activity apps based on MARS dimensions, with the goal of forecasting ratings before production and uncovering insights into user satisfaction drivers.

**Methods:**

We analyzed a dataset of 155 MARS-rated physical activity apps with user ratings. The dataset was split into training (n=111) and testing (n=44) subsets. K means clustering was applied to the training data, identifying 2 clusters. Exploratory data analysis included box plots, summary statistics, and component+residual plots to visualize linearity and distribution patterns across MARS dimensions. Correlation analysis was performed to quantify relationships between each MARS dimension and user ratings. In total, 5 machine learning models—generalized additive models, k-nearest neighbors, random forest, extreme gradient boosting, and support vector regression—were trained with and without clustering. Models were hypertuned and trained separately on each cluster, and the best-performing model for each cluster was selected. These predictions were combined to compute final performance metrics for the test set. Performance was evaluated using correct prediction percentage (0.5 range), mean absolute error, and *R*². Validation was performed on 2 additional datasets: mindfulness (n=85) and older adults (n=55) apps.

**Results:**

Exploratory data analysis revealed that apps in cluster 1 were feature-rich and scored higher across all MARS dimensions, reflecting comprehensive and engagement-oriented designs. In contrast, cluster 2 comprised simpler, utilitarian apps focused on basic functionality. Component+residual plots showed nonlinear relationships, which became more interpretable within clusters. Correlation analysis indicated stronger associations between user ratings and engagement and functionality, but weaker or negative correlations with aesthetics and information, particularly in cluster 2. In the unclustered dataset, k nearest neighbors achieved 79.55% accuracy, mean absolute error=0.26, and *R*²=0.06. The combined support vector regression (cluster 1)+k-nearest neighbors (cluster 2) model achieved the highest performance: 88.64% accuracy, mean absolute error=0.27, and *R*²=0.04. Clustering improved prediction accuracy and enhanced alignment between predicted and actual user ratings. Models also generalized well to the external datasets.

**Conclusions:**

The combined clustering and modeling approach enhances prediction accuracy and reveals how user satisfaction drivers vary across app types. By transforming MARS from a descriptive tool into a predictive framework, this study offers a scalable, transparent method for forecasting user ratings during app development—particularly useful in early-stage or low-data settings.

## Introduction

The Mobile App Rating Scale (MARS) is a tool designed to assess the quality of mobile health apps [1]. It was developed to meet the increasing demand for a standardized and reliable method for app evaluation. This 23-item, 5-point rating system evaluates aspects such as user engagement (engagement), feature performance (functionality), visual design (aesthetics), content accuracy (information), and overall satisfaction, providing a holistic assessment to identify high-quality apps [2].

Although MARS has been widely used to quantify app quality across various categories [3-11], no study has yet explored its potential to predict app user ratings—a key indicator of an app’s popularity and perceived value. User ratings are meaningful because they reflect the aggregated experiences of the user base and often influence app visibility, downloads, and credibility in app store ecosystems. Exploring how MARS scores relate to these ratings could offer insights into the underlying drivers of user satisfaction and support more informed design and evaluation decisions.

This work builds upon our previous research in which we applied k-means clustering to MARS-rated drug-drug interaction apps [12]. That study revealed that the correlation between MARS dimensions and user ratings varied significantly across app clusters. In some groups, functionality was most strongly associated with user ratings, while in others, dimensions like engagement or information played a larger role. These findings highlighted that relationships between expert scores and user feedback are not uniform but highly context-dependent. Clustering enabled the identification of feature-based subgroups aligned with distinct satisfaction profiles—reinforcing the value of segmentation when interpreting MARS-based evaluations.

Complementary work by Aziz et al [13] and Mertens et al [14] also demonstrated the value of clustering in understanding user behavior and engagement in mobile interventions. Aziz et al identified behavioral archetypes based on app usage and personality traits, while Mertens et al showed that user impact varies depending on engagement profiles over time. Although these studies focus on clustering users rather than apps, they reinforce a common insight: segmentation enhances our understanding of satisfaction drivers.

Alternatively, other researchers have pursued bottom-up strategies for quality assessment that rely on postlaunch user data. For example, Haoues et al [15] used machine learning and natural language processing on user reviews within the International Organization for Standardization or International Electrotechnical Commission 25010 framework to assess app quality. While these methods can capture sentiment and usability themes, they require large volumes of user feedback—often unavailable in early-stage development.

Building on these findings, our study combines unsupervised clustering and supervised machine learning to predict app store user ratings based on expert-evaluated app quality dimensions. By segmenting apps into meaningful groups and training cluster-specific models, we aimed to improve prediction accuracy and uncover which MARS dimensions matter most in each context. This top-down approach enables forecasting of user satisfaction before release, making it particularly valuable in low-data settings. Ultimately, it offers developers a proactive, scalable framework to anticipate user expectations and optimize app design before launch.

## Methods

### Reporting Standards

This study adheres to the Transparent Reporting of a multivariable prediction model for Individual Prognosis or Diagnosis (TRIPOD) [16] guidelines to ensure comprehensive and transparent reporting of prediction model development and evaluation.

### Data Source and Eligibility Criteria

**Figure 1.**
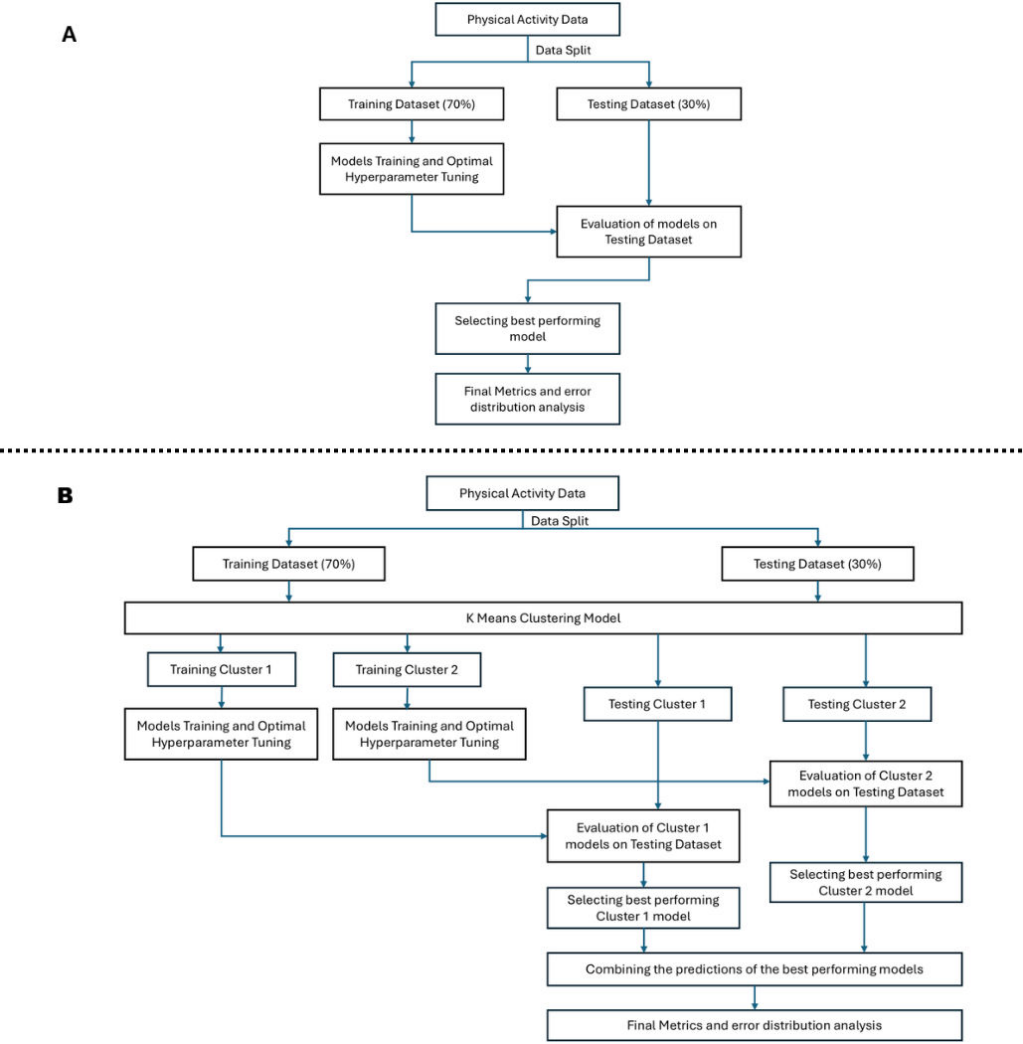
Workflow for predictive modeling (1A) without clustering (1B) with K-means clustering.

This study uses physical activity app data originally compiled by Paganini et al [3] and made accessible by Terhorst et al [17]. The dataset included item-level MARS scores previously evaluated and published by the original authors as part of their systematic app evaluation studies, with complete assessments by trained experts. No new MARS evaluations were conducted. Since the objective of this study was to examine the predictive utility of MARS scores in relation to user ratings, we worked directly with the original reported scores to maintain analytical neutrality and avoid introducing evaluator bias. Of the 312 apps in the dataset, 155 had complete MARS evaluations and corresponding user ratings and were included in the analysis. Apps with missing data for any dimension or user rating were excluded using a complete-case approach, with no imputation performed. Figure 1 illustrates the overall workflow of the study, with Figure 1A depicting the modeling process without clustering and Figure 1B showing the process incorporating clustering.

### Outcome and Predictor Variables

The outcome variable was the average user rating on a scale from 1 to 5. Predictor variables included the 4 MARS dimensions: engagement, functionality, aesthetics, and information. No transformations or standardization were applied to the predictor variables.

### Exploratory Data Analysis

The dataset was first divided into training (111/155, 70%) and testing (44/155, 30%) sets. The training dataset was used for exploratory data analysis (EDA) and training the k-means and prediction models, while the testing dataset was held out for final model evaluation. During the EDA phase, we examined the relationships between user ratings and the 4 MARS dimensions. We visualized distribution patterns using box plots, which provided insights into the central tendencies and variability across the dimensions. In addition, we created a summary table to present descriptive statistics for each dimension, including the mean, median, 25th and 75th percentiles, range, minimum, and maximum values [12,18]. We then generated component+residual plots for each MARS dimension to better understand the data patterns and guide the selection of appropriate modeling techniques [19]. In these plots, the blue line represents the fitted values showing the main trend between the predictor and user ratings, while the pink line provides a smoother fit, highlighting any deviations or nonlinear relationships. Finally, we conducted a correlation analysis to quantify the strength and direction of associations between each MARS dimension and user ratings, highlighting dimensions with the most significant impact on user satisfaction [12].

### Model Development and Hyperparameter Tuning

We applied 5 machine learning models: generalized additive models (GAM), k-nearest neighbors (KNN), random forest (RF), extreme gradient boosting (XGBoost), and support vector regression (SVR). Each model was selected for its unique approach to capturing different data patterns: GAM for handling potential nonlinear relationships [20], KNN for proximity-based prediction by averaging ratings of similar apps [21], RF as an ensemble approach combining multiple decision trees to improve accuracy [22], XGBoost for its boosting techniques that enhance prediction accuracy [23], and SVR for determining the optimal hyperplane to separate groups effectively [24].

Hyperparameter tuning for each of the 5 models was conducted using 5-fold cross-validation on the training dataset [25], optimizing the models for the best predictive performance. For GAM, tuning involved adjusting the number of knots in the smoothing splines to capture different levels of nonlinearity [26]. For KNN, a range of k values was evaluated to find the optimal number of neighbors [27]. For RF, the number of randomly selected features at each split was tuned to balance model depth and generalization [23]. For XGBoost, the number of boosting rounds, learning rate, and tree depth were optimized [23]. SVR tuning involved testing different combinations of cost and gamma parameters to optimize the margin of tolerance and the influence of individual data points [24]. The best hyperparameters were selected based on achieving the highest percentage of correct predictions (within a 0.5 range), the lowest mean absolute error (MAE), and the highest *R*^2^ values [28]. After identifying the optimal hyperparameters, the models were evaluated on the testing dataset using the same metrics. All analyses were conducted using the latest version of RStudio.

### Clustering Analysis

To further refine the predictive modeling, we incorporated k-means clustering as a preprocessing step, grouping the apps into clusters based on their MARS scores. This approach aimed to capture more nuanced relationships between app characteristics and user ratings [12]. The optimal number of clusters was determined using an elbow plot, which identifies the point where the within-cluster sum of squares (WCSS) shows a diminishing return with increasing clusters [12,29]. Although widely used, this method involves a degree of subjectivity, as identifying the inflection point in the curve relies on visual interpretation.

### Cluster-Specific Modeling

To characterize the clusters identified through k-means, we performed a second round of EDA, mirroring the initial steps conducted on the entire training dataset. Following the EDA, the machine learning models were applied separately to each cluster, with hyperparameter tuning conducted individually for each cluster to ensure optimal model performance. This cluster-specific approach aimed to better capture the unique relationships between MARS scores and user ratings within each group.

The testing dataset was segmented using the trained k-means clustering model to allocate each data point to the appropriate cluster. Each cluster-specific model was then tested on its respective cluster within the testing dataset, using the same evaluation metrics as before.

### Combined Prediction and Performance Evaluation

To assess the overall effectiveness of the clustering-based approach, predictions from the best-performing models were consolidated to compute the overall metrics for the entire testing dataset. The best model for each cluster was selected based on the highest correct prediction percentage, the lowest MAE, and the highest *R*^2^ value. Predictions from each cluster’s best model were combined to calculate the final overall metrics for the entire testing dataset. This enabled the direct comparison of outcomes of the model’s performance with and without the clustering step.

Scatter plots of actual versus predicted user ratings, with a 0.5 tolerance band, were used to visually assess alignment across the test set for both unclustered and clustered models. The diagonal line in each plot indicates where predicted ratings equal actual ratings. We also analyzed the distribution of prediction accuracy across error ranges, which provided deeper insights into how many apps were accurately predicted within each range. These distributions were visualized using bar plots.

### Model Generalizability Across App Categories

To validate our model’s generalizability, we applied the same clustering and modeling approach to 2 additional datasets: mindfulness [4] and older adults [6] apps. The process of evaluation remained the same, with clustering, hyperparameter tuning, and model evaluation performed as previously described.

### Ethical Considerations

This study did not involve human participants, patient data, or identifiable information. The analysis was limited to publicly available mobile applications and datasets. Institutional review board approval was not required, and informed consent was not applicable.

## Results

### K Means Clustering

**Figure 2.**
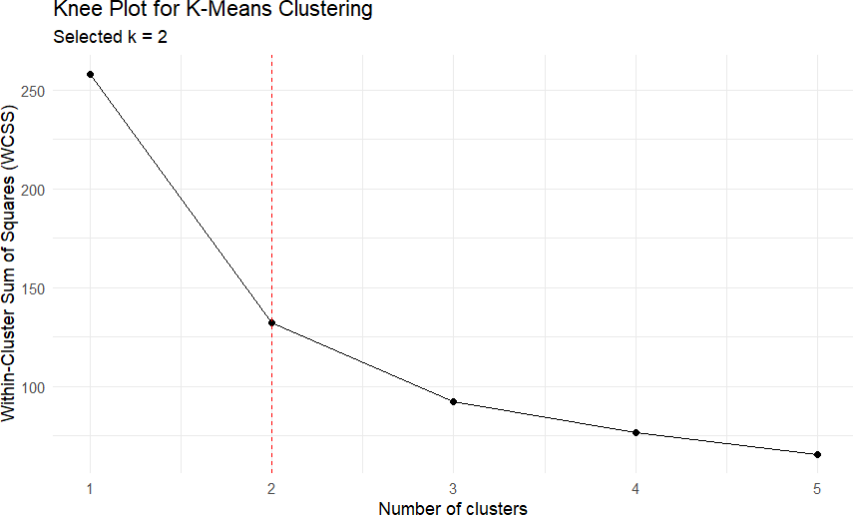
Knee plot for K means clustering showing the Within-Cluster Sum of Squares (WCSS) against the number of clusters.

The knee plot, shown in Figure 2, illustrates the relationship between the number of clusters and the WCSS. As the number of clusters increases, the WCSS decreases, indicating that data points are more tightly grouped within each cluster [12]. However, beyond 2 clusters, the rate of decrease in WCSS slows significantly, marking an “elbow” point. This suggests that adding more clusters beyond 2 provides diminishing returns in compactness. Thus, selecting 2 clusters was deemed optimal, as it balances the trade-off between data cohesion within clusters and the simplicity of the model structure.

### EDA

#### Data Distribution

**Figure 3.**
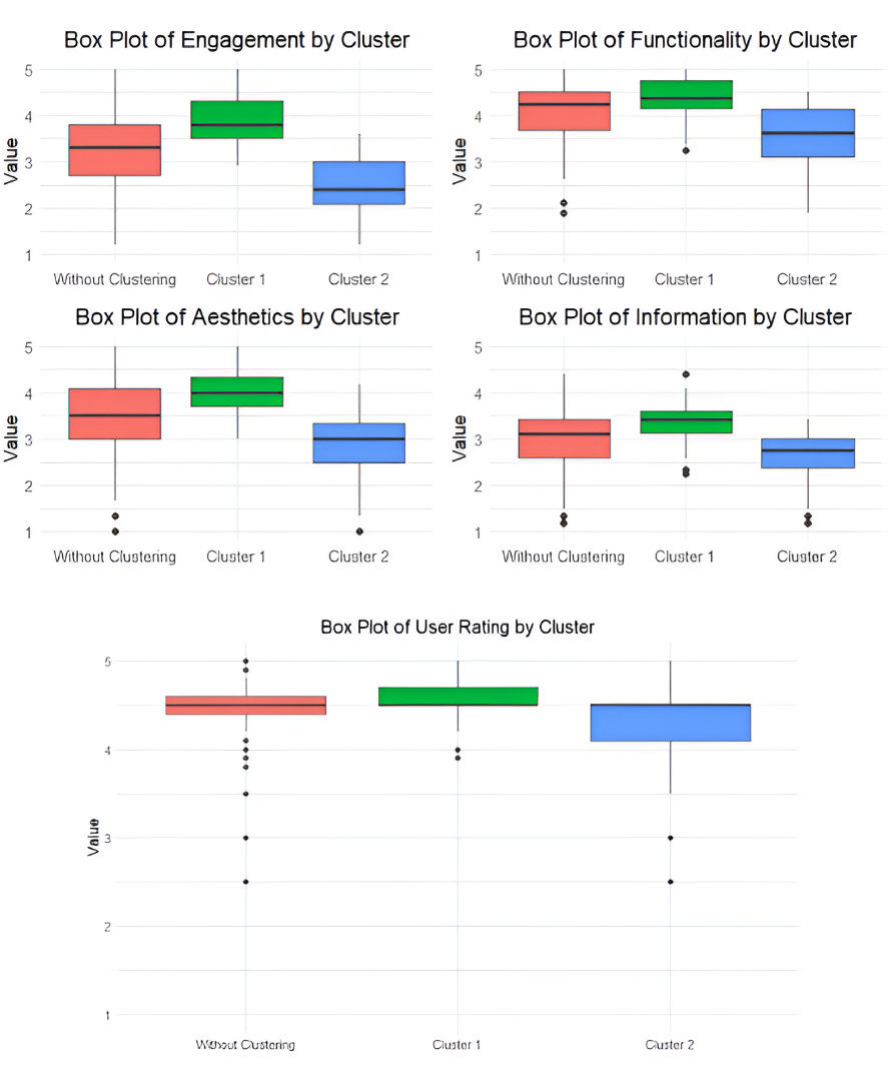
Box plots of MARS dimensions and user ratings across unclustered and clustered data. MARS: Mobile App Rating Scale.

The EDA began by examining the distributions of the 4 MARS dimensions and user ratings without applying clustering. The box plots (Figure 3) for the “Without Clustering” group showed centralized distributions across all dimensions, with some variability, particularly in engagement (range=3.80) and aesthetics (range=4.00). These findings provided a baseline understanding of the training dataset’s characteristics.

Application of k-means clustering resulted in 2 clusters: cluster 1 with 62 apps (55.9%) and cluster 2 with 49 apps (44.1%). The trained clustering model was then used to assign the testing apps to clusters, resulting in 21 apps in cluster 1 and 23 in cluster 2. Although the distribution is slightly imbalanced, both clusters retained sufficient size in both the training and testing sets to support reliable model training and evaluation. We then re-evaluated the distributions for each dimension and user rating within each cluster, as shown in the box plots (Figure 3) and summary statistics table (Table 1). Cluster 1 consistently exhibited higher medians across all dimensions—engagement (3.80), functionality (4.38), aesthetics (4.00), and information (3.42)—compared to cluster 2, which had corresponding medians of 2.40, 3.62, 3.00, and 2.75, respectively. These results suggest that cluster 1 apps, based on MARS evaluations, demonstrate stronger quality features than cluster 2, which includes lower-rated apps. The “Without Clustering” analysis produced average scores that fell between the 2 clusters.

**Table 1.** Summary statistics for MARS dimensions and user ratings across unclustered and clustered data.

Clusters and Dimensions	Values		
	Mean (SD)	Median (IQR)	Range (Min-Max)
Training data sample size (n=111), without clustering			
Engagement	3.23 (0.90)	3.30 (2.70-3.80)	3.80 (1.20-5.00)
Functionality	4.06 (0.65)	4.25 (3.69-4.50)	3.12 (1.88-5.00)
Aesthetics	3.50 (0.85)	3.50 (3.00-4.09)	4.00 (1.00-5.00)
Information	3.01 (0.61)	3.12 (2.60-3.43)	3.23 (1.17-4.40)
User rating	4.46 (0.37)	4.50 (4.40-4.60)	2.50 (2.50-5.00)
Training data sample size (Cluster 1), (n=62)			
Engagement	3.84 (0.55)	3.80 (3.50-4.30)	2.10 (2.90-5.00)
Functionality	4.42 (0.43)	4.38 (4.15-4.75)	1.75 (3.25-5.00)
Aesthetics	4.05 (0.51)	4.00 (3.71-4.33)	2.00 (3.00-5.00)
Information	3.32 (0.47)	3.42 (3.13-3.60)	2.15 (2.25-4.40)
User ratings	4.56 (0.27)	4.50 (4.50-4.70)	1.10 (3.90-5.00)
Training data sample size (Cluster 2), (n=49)			
Engagement	2.46 (0.61)	2.40 (2.10-3.00)	2.40 (1.20-3.60)
Functionality	3.62 (0.62)	3.62 (3.12-4.12)	2.62 (1.88-4.50)
Aesthetics	2.80 (0.67)	3.00 (2.50-3.33)	3.17 (1.00-4.17)
Information	2.62 (0.55)	2.75 (2.38-3.00)	2.25 (1.17-3.42)
User ratings	4.32 (0.44)	4.50 (4.10-4.50)	2.50 (2.50-5.00)

#### Component+Residual Plots

**Figure 4.**
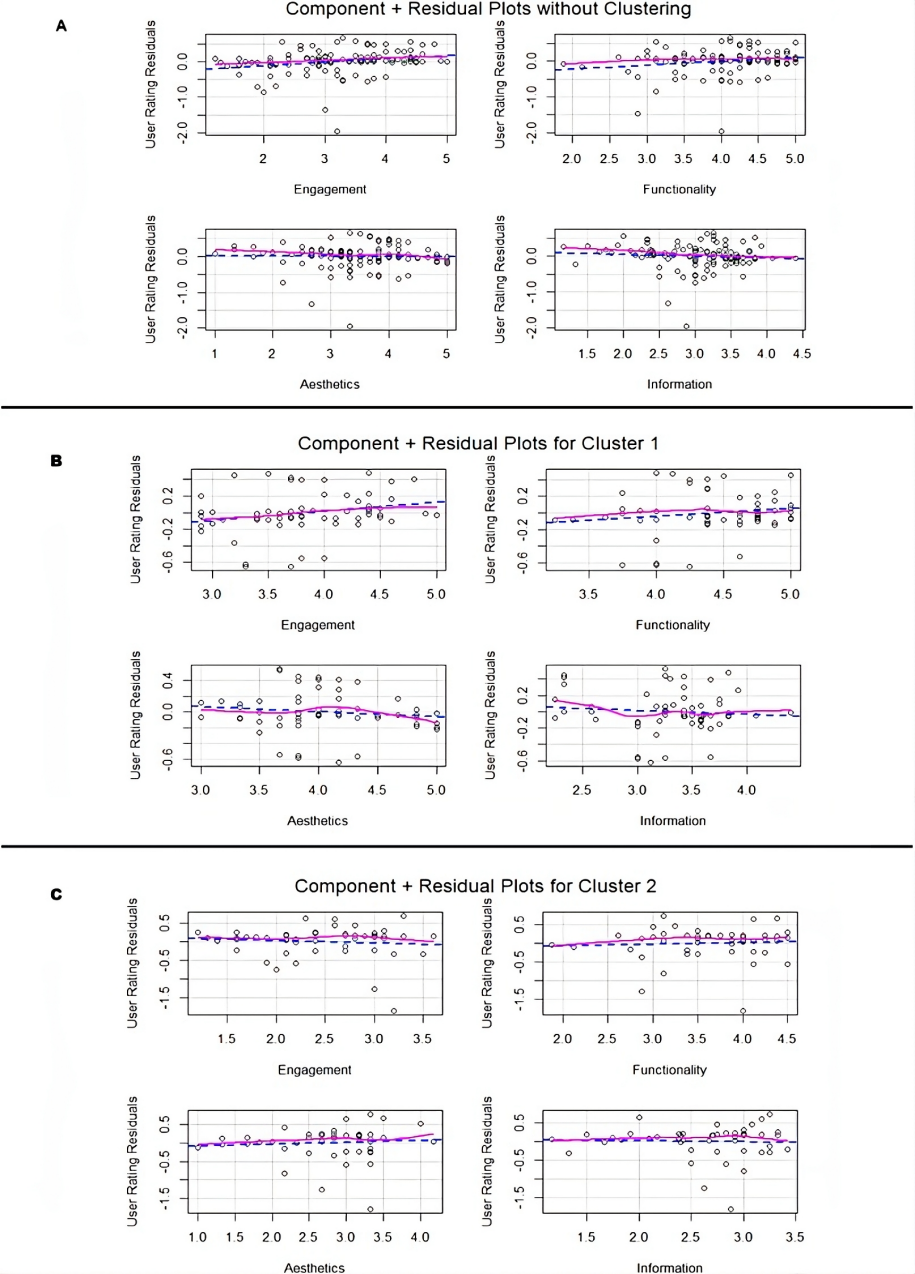
Component+residual plots for MARS dimensions and user ratings (4A) without clustering (4B) for cluster 1 (4C) for cluster 2. MARS: Mobile App Rating Scale

From the component+residual plots of the unclustered analysis (Figure 4A), residuals appear widely dispersed (–2.0 to +1.0) across all dimensions, with only subtle trends. Engagement (1.20 to 5.00) shows a slight positive relationship with user ratings, while functionality (1.88 to 5.00) displays an initial upward trend in residuals up to around 4.0, then plateaus—suggesting diminishing returns at higher scores. In contrast, aesthetics (1.00 to 5.00) and information (1.17 to 4.40) show a negative trend, indicating inverse associations with user ratings. These generalized, nonlinear patterns reflect the broad variability of app characteristics in the training dataset, especially for aesthetics (range=4.00) and engagement (range=3.80).

In Cluster 1 (Figure 4B), residuals were more consistent and compact across all dimensions (–0.6 to +0.4), reflecting the group’s overall uniformity. Engagement (2.90 to 5.00) showed an initial upward trend that plateaued beyond approximately 4.3, while functionality (3.25 to 5.00) also rose early before flattening after 4.5—indicating nonlinear associations with user ratings. Aesthetics (3.00 to 5.00) displayed a complex curve, dipping, then rising, then falling again. Information (2.25 to 4.40) followed a similar nonlinear pattern, declining first, then increasing, and eventually leveling off around 3.5‐4.0. These results suggest that although cluster 1 is more cohesive, relationships between MARS dimensions and user ratings remain complex.

In cluster 2 (Figure 4C), residuals showed greater variability with values ranging from approximately –1.5 to +0.5. Engagement (1.20 to 3.60) showed a downward trend, between 2.0 and 3.2, indicating a weak negative association with user ratings. Functionality (1.88 to 4.50) and aesthetics (1.00 to 4.17) exhibited slight upward trends, between 2.5‐4.0 and 2.0‐3.5, respectively, indicating modest positive associations. Information (1.17 to 3.42) showed a nonlinear pattern—increasing initially up to 2.6, then declining beyond 2.8—suggesting that high Information scores may correspond to lower user ratings. These findings highlight greater heterogeneity and less stable patterns in cluster 2, reinforcing the need for distinct modeling approaches between clusters.

In summary, the unclustered data shows weak and scattered associations. Cluster 1 reveals cohesive but complex nonlinear patterns across all dimensions. Cluster 2 shows more variability, with modest positive trends in functionality and aesthetics.

#### Correlation Analysis

**Figure 5.**
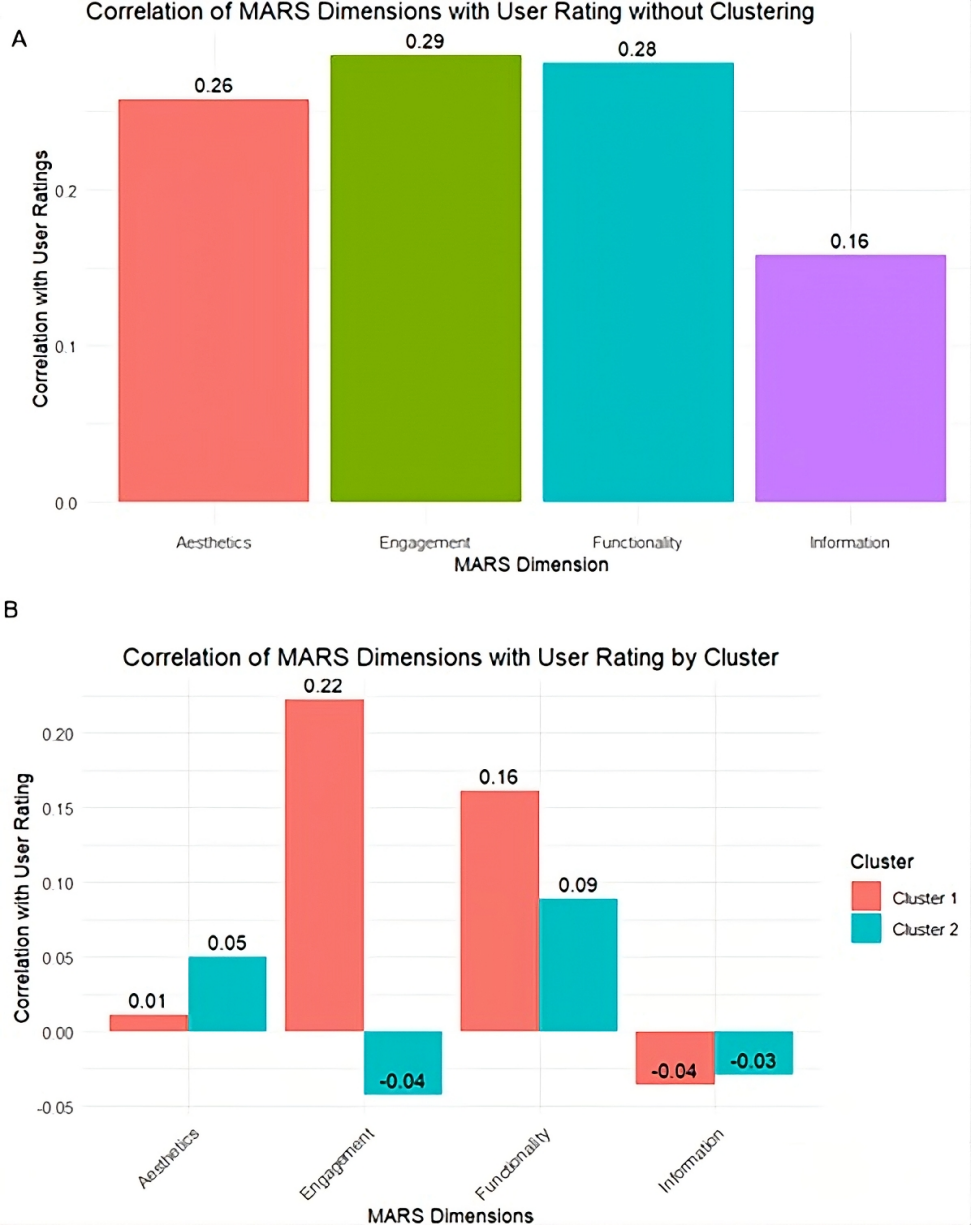
Correlation of MARS dimensions with user rating (5A) without clustering (5B) with clusters. MARS: Mobile App Rating Scale.

Correlation analysis between MARS dimensions and user ratings (Figure 5) revealed different patterns when comparing the unclustered dataset to the segmented clusters. In the unclustered analysis (Figure 5A), engagement and functionality show positive correlations with user ratings, at 0.29 and 0.28, respectively, indicating a moderate association. Aesthetics follows with a correlation of 0.26, while information has the lowest correlation with user ratings at 0.16. These findings suggest that, across the entire dataset, engagement and functionality are the primary drivers of user satisfaction.

Within the clusters (Figure 5B), correlations appear less pronounced. In cluster 1, engagement has the highest correlation (0.22), followed by functionality (0.16). Aesthetics and information show minimal or no correlation, at 0.01 and -0.04, respectively. This indicates that while engagement and functionality remain relevant in cluster 1, their influence is weaker compared to the unclustered data.

In cluster 2, correlations are uniformly weak. Engagement (–0.04) and information (–0.03) show negative correlations, while aesthetics (0.05) and functionality (0.09) exhibit weak positive associations. These findings suggest that in cluster 2, no single MARS dimension strongly influences user ratings, pointing to a user base whose satisfaction may depend on factors outside the scope of MARS, or potential misalignment between expert evaluations and user perceptions.

### Predictions

Table 2 presents the final hyperparameter settings selected for each model and cluster. Table 3 summarizes the predictive performance of the models across the testing dataset and within each cluster.

Without clustering, KNN and SVR achieved the highest correct prediction percentage at 79.55%, both maintaining relatively low MAEs (0.26 and 0.29, respectively). GAM also performed reasonably well with 77.27% accuracy and the lowest MAE (0.25). RF and XGB showed weaker performance, with lower correct prediction percentages (72.72%) and negative *R*^2^ values (−0.19), indicating poor model fit for these models. KNN was selected as the best-performing model without clustering due to its lower MAE of 0.26 and higher *R*-square of 0.06, compared to SVR’s MAE of 0.29 and *R*-square of −0.13.

**Table 2.** Tuned hyperparameters by model type and cluster.

Clusters and models	Selected parameters after hypertuning
Without clustering	
Generalized additive model	Number of spline knots=4
K nearest neighbors	Number of neighbors=7
Random forest	Number of features selected at each split=2
Extreme gradient boosting	Number of boosting rounds=150 Maximum tree depth=1 Learning rate=0.05
Support vector regression	Cost=4 Gamma=4
Cluster 1	
Generalized additive model	Number of spline knots=3
K nearest neighbors	Number of neighbors=7
Random forest nearest neighbors	Number of features selected at each split=4
Extreme gradient boosting	Number of boosting rounds=150 Maximum tree depth=1 Learning rate=0.05
Support vector regression	Gamma=1 Cost=2
Cluster 2	
Generalized additive model	Number of spline knots=3
K nearest neighbors	Number of neighbors=8
Random forest	Number of features selected at each split=2
Extreme gradient boosting	Number of boosting rounds=50 Maximum tree depth=1 Learning rate=0.1
Support vector regression	Cost=0.5 Gamma=4

**Table 3.** Model performance metrics with and without clustering.

Clusters and models	Correct predictions percentage	MAE^a^	*R* ^2^
Without clustering, (n=44)			
GAM^b^	77.27	0.25	0.10
KNN^c^	79.55	0.26	0.06
RF^d^	72.72	0.30	−0.19
XGB^e^	72.72	0.28	−0.19
SVR^f^	79.55	0.29	−0.13
Cluster 1, (n=27)			
GAM	85.19	0.24	0.12
KNN	85.19	0.25	0.14
RF	81.48	0.26	−0.03
XGB	77.78	0.25	0.01
SVR	88.89	0.27	0.05
Cluster 2, (n=17)			
GAM	88.24	0.29	−0.05
KNN	88.24	0.28	0.03
RF	82.35	0.34	−0.45
XGB	64.71	0.43	−1.26
SVR	88.24	0.28	−0.04
Combined cluster model, (n=44) SVR+KNN	88.64	0.27	0.04

a MAE: mean absolute error.

b GAM: generalized additive models.

c KNN: K-nearest neighbors

d RF: random forest.

e XGB: extreme gradient boosting.

f SVR: support vector regression.

In Cluster 1, SVR delivered the highest accuracy, achieving an 88.89% correct prediction rate and an MAE of 0.27. This model was closely followed by GAM and KNN, both achieving 85.19% accuracy, with GAM having a slightly lower MAE (0.24). For RF and XGB, the performance dropped, with RF showing a correct prediction percentage of 81.48% and XGB at 77.78%. The *R*^2^ values indicate a better model fit within this cluster, especially for GAM and KNN, which displayed positive *R*^2^ values, while RF and XGB struggled, showing low or negative values. SVR was selected as the best-performing model for cluster 1.

In cluster 2, SVR, KNN, and GAM achieved the same prediction accuracy, each at 88.24%. RF and XGB showed notable drops in performance, with XGB achieving the lowest accuracy (64.71%) and a high negative *R*^2^ (−1.26), indicating poor fit and low predictive reliability within this cluster. KNN was selected as the best-performing model due to its lower MAE (0.28) and a positive *R*^2^ (0.03), compared to GAM’s MAE of 0.29 and *R*^2^ of −0.05, and SVR’s MAE of 0.28 and *R*^2^ of −0.04.

The best-performing models from each cluster—SVR from cluster 1 and KNN from cluster 2—were selected, and their predictions were combined to compute the final metrics for the entire testing dataset. This combined approach achieved an improved overall correct prediction percentage of 88.64% outperforming the best unclustered data model, KNN, which achieved 79.55% accuracy.

**Figure 6.**
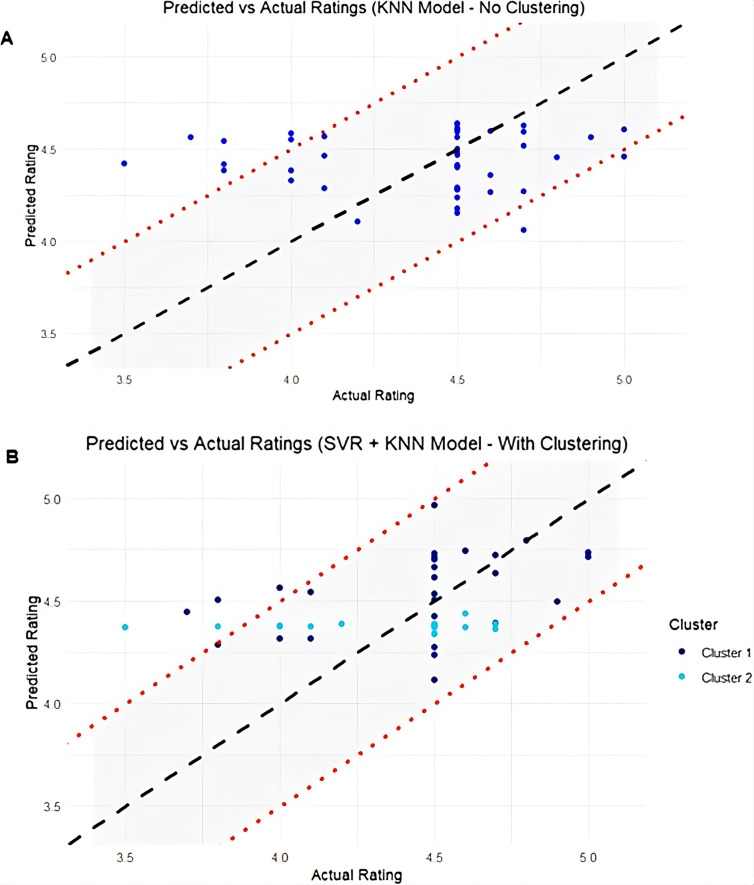
Comparison of actual versus predicted user ratings (6A) KNN model predictions without clustering (6B) Combined SVR and KNN model predictions following clustering. KNN: k-nearest neighbors; SVR: support vector regression.

To evaluate prediction alignment, we plotted actual versus predicted user ratings across the full test set for both the unclustered and clustered approaches (Figure 6). The unclustered KNN model (Figure 6A) exhibited wider deviations, particularly for lower and higher actual ratings. Two apps were underpredicted, and seven were overpredicted. In contrast, the cluster-based SVR+KNN model (Figure 6B) showed closer alignment with the actual values, with a greater number of predictions falling within the 0.5 tolerance band. Five apps were overpredicted, with 3 belonging to cluster 1 and 2 to cluster 2. This indicates improved accuracy and consistency following the clustering step.

**Figure 7.**
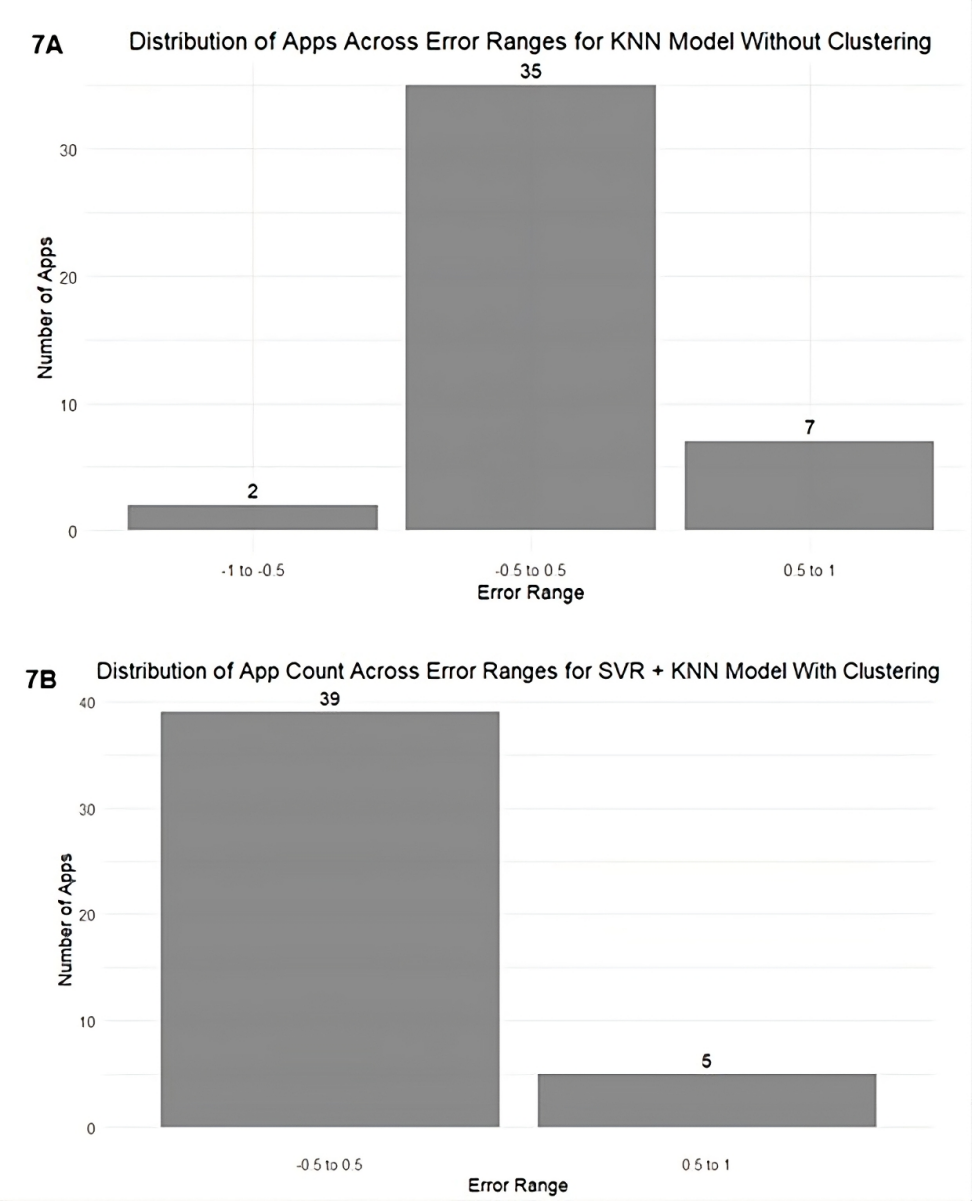
Error distribution of predictions (7A) using KNN model without clustering (7B) using SVR+KNN model with clustering. KNN: k-nearest neighbors; SVR support vector regression.

The error distribution plots (Figure 7) show the extent of prediction deviations across models. In the unclustered KNN model (Figure 7A), 2 apps had errors between −1 to −0.5, 35 were within the −0.5 to 0.5 range, and 7 had errors between 0.5 to 1. In comparison, the clustered SVR+KNN model (Figure 7B) showed improved precision, with 39 apps within the −0.5 to 0.5 range and only 5 apps falling in the 0.5 to 1 range.

Further to assess generalizability, the clustering-based modeling framework was validated on 2 external datasets: mindfulness apps (n=85) and older adult apps (n=55). In both cases, clustering either matched or outperformed the best-performing unclustered models. For the mindfulness dataset, the combined SVR+XGB cluster model achieved the highest overall accuracy (66.67%), a lower MAE (0.46), and a positive *R*² (0.07)—improving upon the best unclustered model (SVR), which had a negative *R*² (–0.06). Notably, cluster 2 models outperformed cluster 1, with XGB achieving an *R*² of 0.40, indicating that meaningful patterns emerged when segmenting the apps. For the Elderly dataset, the combined KNN-based cluster model produced the best results, with 73.34% accuracy, an MAE of 0.48, and a positive *R*² (0.12)—surpassing the unclustered RF model, which had an *R*² of –0.08. Full results of both datasets are detailed in the Multimedia Appendix 1.

## Discussion

### Principal Findings

This study evaluated whether MARS scores could be used to predict user ratings through a clustering-based machine learning approach. The combined SVR+KNN model outperformed unclustered models, with higher accuracy and lower error. Clustering revealed distinct satisfaction patterns across app types and improved prediction alignment. These findings suggest that expert-driven MARS evaluations, when paired with data-driven modeling, can effectively forecast user satisfaction and ratings before app release.

In the unclustered dataset, MARS scores tended to average out, potentially obscuring the influence of specific features. Clustering enabled a deeper examination by isolating distinct subgroups, revealing more nuanced relationships between app characteristics and satisfaction—reflecting the diverse feature priorities among physical activity app users.

Cluster 1, characterized by higher mean scores across all MARS dimensions, appears to represent apps that align closely with user expectations by delivering a balanced, comprehensive experience. Users in this cluster may value features such as personalized workout recommendations, progress tracking, interactive challenges, or goal-setting tools [30-33]. These attributes contribute to a well-rounded app experience, where multiple dimensions—such as engagement through interactive features and functionality through reliable tracking—enhance overall satisfaction as reflected in the correlation analysis (Figure 5) with user ratings.

Conversely, cluster 2 exhibited lower scores across all MARS dimensions, likely representing simpler, function-focused apps. Residual plots (Figure 4C) showed negative patterns for engagement and information and modest positive trends for functionality and aesthetics—indicating that only certain dimensions influenced user ratings. Correlation analysis (Figure 5B) further supported this, revealing weak or negative associations for most dimensions, with functionality showing the strongest correlation. These patterns suggest that users in this segment prioritize basic features such as step counting, activity logging, or workout timers, with less emphasis on visual design or interactive elements [30-33].

Notably, the median user rating for the unclustered dataset, as well as for both clusters, remained consistent at 4.5 (Table 1), indicating a shared baseline of high satisfaction across groups, despite the lower MARS dimension scores observed in cluster 2. This indicates that functional adequacy alone may drive satisfaction for utilitarian apps, even when MARS scores are lower. This observation aligns with previous research showing that user expectations vary by app context. For example, Portenhauser et al [6] found that older adults prioritize simplicity, while Mani et al [4] noted that mindfulness app users emphasize content credibility. These findings also resonate with our earlier work on drug-drug interaction apps [12], which showed that the relationship between MARS dimensions and user satisfaction varied across clusters. Collectively, these results reinforce that no single MARS dimension universally predicts user satisfaction; instead, the relevance of each dimension depends on the app’s functional scope and the expectations of its target audience.

From the correlation analysis, we observe that only certain MARS dimensions—particularly engagement and functionality—consistently correlate with user ratings, though the strength of these associations varies by cluster. This raises the broader question of how well expert evaluations align with real-world user perceptions [34,35]. While MARS is designed to objectively assess app quality, our findings suggest that its dimensions do not contribute equally to user satisfaction. For example, in cluster 2, all dimensions show weak or negligible correlations, offering little explanatory value regarding what drives user satisfaction in this group. This underscores the importance of context-aware evaluations—an insight that only became apparent after clustering revealed how user expectations vary across app groups.

In predictive modeling, KNN emerged as the top performer in the unclustered dataset, achieving an accuracy of 79.55%, with a relatively low MAE of 0.26 and an *R*^2^ of 0.06. The low MAE suggests that KNN’s predictions were generally close to actual user ratings, but the modest *R*^2^ reflects limited explanatory power.

The prediction outcomes improved when clustering was applied, as models could be tailored to more similar groups of apps. In Cluster 1, SVR achieved the highest accuracy at 88.89%, with an MAE of 0.27 and a modest *R*^2^ of 0.05, while KNN and GAM also showed improved performance with *R*^2^ values above 0.10. This suggests that in apps with higher MARS scores, expert-assessed quality tends to align more closely with what users value—which is reflected in the improved *R*^2^ values.

In contrast, Cluster 2 posed greater challenges. Although KNN and SVR both achieved high accuracy (88.24%), *R*^2^ values for most models remained low or negative—notably −1.26 for XGB and –0.45 for RF. Even the best-performing models in this cluster, like KNN, achieved only a 0.03 *R*^2^, indicating that while predictions were accurate in terms of MAE, they explained very little of the variance in user ratings. This suggests that user satisfaction in these simpler apps may stem from minimal but sufficient functionality rather than broader quality dimensions emphasized by MARS. The weak positive correlation for functionality further supports this interpretation, highlighting a possible disconnect between expert-assessed quality and user-perceived value in utility-focused apps.

The combined approach—using SVR for cluster 1 and KNN for cluster 2—achieved the highest overall accuracy at 88.64%, with a consistent MAE of 0.27 and *R*^2^ of 0.04. While this confirms the value of clustering, the modest *R*^2^ values across both settings underscore that MARS alone cannot fully explain user ratings. User satisfaction may be influenced by external factors such as marketing, demographics, or social dynamics—elements not captured by MARS. This highlights the need for future studies to incorporate contextual and user-centered variables (eg, downloads and review sentiment) to improve generalizability.

Further, an interesting observation emerged that even though the correlations between individual MARS dimensions and user ratings were weaker within clusters, the prediction accuracy improved. This highlights a limitation of traditional correlation analysis, which only captures simple, one-to-one relationships. In contrast, machine learning models can identify more complex patterns by analyzing multiple variables together, especially after clustering separates apps into more similar groups. This explains why weak correlations can still lead to strong predictions—an effect also noted in previous research that supports using model-based approaches over basic correlation in complex data contexts [36,37].

The comparison of actual versus predicted ratings shows that the unclustered KNN model (Figure 6A) exhibited underfitting, particularly at the extremes—likely a result of averaging across diverse app types. In contrast, the clustered SVR+KNN model (Figure 6B) demonstrated closer alignment with actual ratings and fewer large deviations. This improvement can be attributed to clustering, which grouped similar apps together, enabling the models to learn clearer and more relevant patterns. The error distribution plots (Figure 7) further support this, showing a reduction in the number and extent of prediction errors. Importantly, the overpredicted apps in the SVR+KNN model were distributed across both clusters—3 from cluster 1 and 3 from cluster 2—indicating that residual errors were not driven by any single subgroup but instead reflect natural prediction variability.

The evaluation of our framework on 2 external datasets—mindfulness and older adults—further supports the model’s generalizability. As reported in the Results, clustering-based models consistently outperformed or matched the best unclustered models across both domains.

Taken together, our findings highlight the value of integrating structured expert evaluations with unsupervised clustering and machine learning to forecast user satisfaction in mobile health apps. By transforming MARS scores into actionable predictors, this framework enables early-stage evaluation of app quality, especially in the absence of large-scale user feedback. The improved predictive performance of cluster-specific models—validated across both physical activity and external domains—demonstrates that user satisfaction is best understood as context-specific, shaped by both app features and audience expectations. As the mobile health ecosystem continues to expand, such scalable and interpretable modeling approaches can play a pivotal role in guiding user-centered app design, regulatory review, and quality benchmarking before release. For real-world implementation, developers can first assign MARS scores to a representative set of apps within their domain to build context-specific clusters. They can then score their own app using the MARS framework and input these scores into our modeling pipeline to forecast its projected user rating. In future work, we aim to develop a standardized, user-friendly tool that will allow developers and evaluators to input MARS scores and receive predicted user satisfaction outcomes, streamlining early-stage decision-making.

### Conclusion

This study demonstrates the effectiveness of combining unsupervised k-means clustering with supervised machine learning models to predict user ratings based on MARS dimensions in physical activity apps. The clustering approach revealed distinct user segments—cluster 1 users preferred feature-rich, engaging apps, while cluster 2 users gravitated toward simpler, utility-focused tools. By tailoring models to these groups, our combined approach (SVR for cluster 1 and KNN for cluster 2) outperformed the best unclustered model (KNN) in accuracy and reliability, offering more context-aware and consistent predictions.

This framework enables developers to forecast user satisfaction before app release using MARS-based expert evaluations. By identifying their app’s alignment with specific user clusters, teams can make informed design choices, benchmark against competitors, and tailor strategies—even in low-data environments.

While prior applications of MARS have largely been descriptive, this study presents a predictive framework that transforms expert scores into actionable, prelaunch indicators of user satisfaction. Unlike postmarket sentiment analysis, our method supports proactive, data-informed decision-making that bridges the gap between expert evaluation and real-world user expectations.

By demonstrating improved predictive performance across clusters and validating the approach on external datasets, this work lays the foundation for a new generation of app evaluation tools that are not only interpretable and resource-efficient but also predictive. Future research should build on this by incorporating richer behavioral signals and contextual app metadata to further improve personalization and model precision.

### Limitations

This study has several limitations. First, MARS scores offer a static, expert-driven evaluation and may not capture changes in app quality over time. Second, user satisfaction is shaped by contextual and behavioral factors that are not included in the MARS framework. Third, the sample size may limit the robustness of clustering and reduce generalizability across broader app ecosystems. Fourth, the accuracy of predictions is contingent on high-quality and consistent MARS scoring, as variability in rating practices could undermine model performance. Fifth, there is a risk of overfitting, particularly within cluster-specific models trained on relatively small subsets. Although hyperparameter tuning and cross-validation were used to minimize this, models such as RF and XGB may still capture spurious patterns specific to the training data. Finally, while the models were hypertuned using cross-validation, tuning choices are often context-dependent, and future applications may require re-optimization to fit specific app categories or user groups.

### Future Scope of Work

Future work will be to quantify how well expert-driven MARS evaluations align with real-world user ratings, especially across diverse user segments. Incorporating additional contextual data—such as user demographics, review sentiment, or app usage patterns—may help explain residual variance and improve model generalizability beyond MARS dimensions alone. To support practical adoption, we also plan to develop a standardized, user-friendly tool that enables developers to input MARS scores and apply our modeling framework to forecast projected user ratings, thereby streamlining early-stage decision-making.

## Supplementary material

10.2196/70987 Multimedia Appendix 1 Validation of model using mindfulness data.

10.2196/70987 Checklist 1 TRIPOD checklist.

## Abbreviations

EDA: exploratory data analysis
GAM: generalized additive models
KNN: k-nearest neighbors
MAE: mean absolute error
MARS: Mobile App Rating Scale
RF: random forest
SVR: support vector regression
TRIPOD: Transparent Reporting of a multivariable prediction model for Individual Prognosis or Diagnosis
WCSS: within-cluster sum of squares
XGB: extreme gradient boosting
