# Supplementary material for: Cluster-Based Predictive Modeling of User Ratings for Physical Activity Apps Using Mobile App Rating Scale (MARS) Dimensions: Model Development and Validation
Source: JMIR Mhealth Uhealth. 2025 Nov 6;13:e70987. doi: 10.2196/70987 (PMC12599983; doi:10.2196/70987)
Supplement: Multimedia Appendix 1 [file mhealth-v13-e70987-s001.docx]

**Validation of Model using Mindfulness Data**

1. **K Means Clustering**

The Mindfulness dataset, provided by Mani et al. [1], contains 85 apps with available user ratings. This dataset was divided into 70% training (n=61) and 30% testing (n=24) subsets. The knee plot analysis [Figure 1] determined that two clusters provide the optimal segmentation.


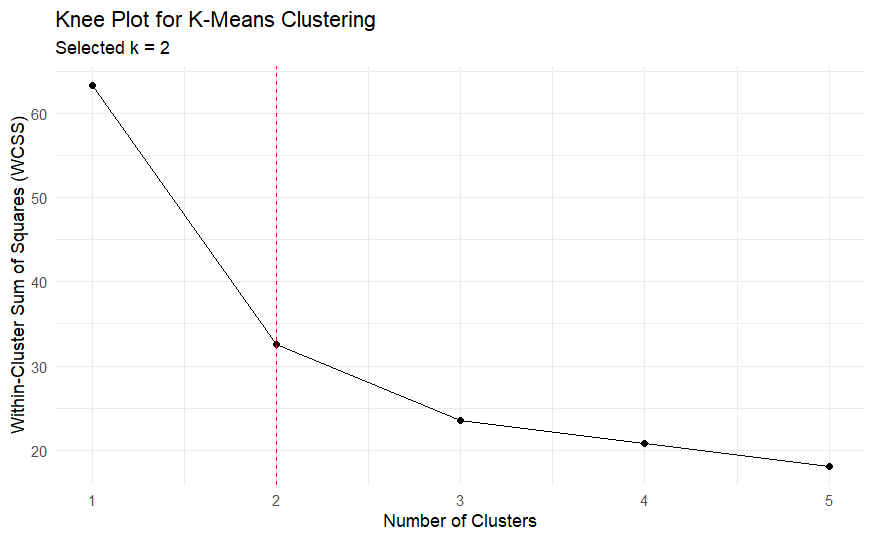


**Figure 1:** Knee Plot for K-Means Clustering showing the Within-Cluster Sum of Squares (WCSS) against the number of clusters for Mindfulness Data

1. **Data Distribution**

The summary statistics table [Table 1] highlights distinct differences between the unclustered dataset and the two clusters, each exhibiting unique patterns across MARS dimensions and User Ratings.

In the unclustered data, mean values of dimensions appear moderate, reflecting an overall balance but without distinguishing specific trends. The user rating median remains high at 4.5, suggesting a general satisfaction but lacking detailed segmentation.

Cluster 1, with lower mean scores across all MARS dimensions compared to the unclustered data, shows a trend toward more basic app functionality. This is accompanied by a slightly lower average user rating, indicating that while these apps meet essential standards, they do not emphasize attributes that might drive higher satisfaction.

Cluster 2 shows higher averages across dimensions, with a consistently high user rating median of 4.5, suggesting that this cluster consists of apps offering richer features and greater overall quality. The elevated scores in each dimension indicate a stronger alignment with higher-rated features.

**Table 1:** Summary Statistics for MARS Dimensions and User Ratings Across Unclustered and Clustered Data for Mindfulness Data

| **Cluster** | **Training Data Sample Size (n)** | **Dimension** | **Mean** | **Median** | **25th Percentile** | **75th Percentile** | **Range** | **Min** | **Max** |
| --- | --- | --- | --- | --- | --- | --- | --- | --- | --- |
| **Without Clustering** | 61 | **Engagement** | 3.42 | 3.30 | 3.00 | 3.90 | 2.50 | 2.20 | 4.70 |
|  |  | **Functionality** | 4.03 | 4.00 | 3.75 | 4.25 | 2.00 | 3.00 | 5.00 |
|  |  | **Aesthetics** | 3.74 | 3.67 | 3.33 | 4.17 | 2.33 | 2.50 | 4.83 |
|  |  | **Information** | 3.08 | 3.10 | 2.75 | 3.42 | 2.08 | 2.00 | 4.08 |
|  |  | **User Rating** | 4.25 | 4.50 | 4.00 | 4.50 | 3.00 | 2.00 | 5.00 |
| **1** | 39 | **Engagement** | 3.05 | 3.10 | 2.85 | 3.30 | 1.80 | 2.20 | 4.00 |
|  |  | **Functionality** | 3.86 | 3.88 | 3.62 | 4.12 | 1.38 | 3.00 | 4.38 |
|  |  | **Aesthetics** | 3.49 | 3.50 | 3.25 | 3.83 | 1.67 | 2.50 | 4.17 |
|  |  | **Information** | 2.85 | 2.90 | 2.50 | 3.18 | 1.70 | 2.00 | 3.70 |
|  |  | **User Ratings** | 4.10 | 4.50 | 4.00 | 4.50 | 3.00 | 2.00 | 5.00 |
| **2** | 22 | **Engagement** | 4.09 | 4.10 | 3.80 | 4.30 | 1.30 | 3.40 | 4.70 |
|  |  | **Functionality** | 4.33 | 4.25 | 4.00 | 4.50 | 1.12 | 3.88 | 5.00 |
|  |  | **Aesthetics** | 4.18 | 4.17 | 4.00 | 4.33 | 1.16 | 3.67 | 4.83 |
|  |  | **Information** | 3.49 | 3.50 | 3.35 | 3.65 | 1.15 | 2.93 | 4.08 |
|  |  | **User Ratings** | 4.50 | 4.50 | 4.50 | 4.50 | 1.00 | 4.00 | 5.00 |

1. **Predictions**

For the unclustered dataset, SVR achieved the highest prediction accuracy at 66.67%, with a MAE of 0.49 and an R-squared value of -0.06 [Table 2].

In Cluster 1, predictive performance was slightly lower, likely reflecting the cluster's lower scores across MARS dimensions and user ratings. SVR led again, with a 61.54% correct prediction rate, an MAE of 0.60, and a slightly negative R-squared of -0.07. The performance across models in this cluster generally showed lower prediction accuracy and higher MAEs, suggesting that user ratings for apps in Cluster 1 were harder to predict accurately.

Cluster 2 demonstrated improved predictive performance compared to Cluster 1, with all models achieving a correct prediction rate of 72.73%. XGB stood out, achieving the lowest MAE of 0.30 and the highest R-squared of 0.40, indicating that it was best at capturing user rating variance in this group.

The combined cluster model, which used SVR for Cluster 1 and XGB for Cluster 2, achieved an overall accuracy of 66.67%, with an MAE of 0.46 and an R-squared of 0.07. This approach outperformed the best unclustered model in terms of error reduction, as the combined model’s MAE was slightly lower, indicating better consistency in predicting user ratings across the two distinct user segments. The positive R-squared value further suggests an improvement in the model’s reliability when tailored to each cluster.

**Table 2:** Model Performance Metrics with and without clustering for Mindfulness data

| **Cluster** | **Testing Data Sample Size (n)** | **Model** | **Correct Predictions Percentage** | **MAE** | **R Squared** |
| --- | --- | --- | --- | --- | --- |
| Without Clustering | 24 | GAM | 62.50 | 0.51 | 0.22 |
|  |  | KNN | 62.50 | 0.48 | -0.05 |
|  |  | RF | 50.00 | 0.54 | -0.07 |
|  |  | XGB | 62.50 | 0.49 | 0.05 |
|  |  | SVR | 66.67 | 0.49 | -0.06 |
| Cluster 1 | 13 | GAM | 46.15 | 0.61 | 0.25 |
|  |  | KNN | 53.85 | 0.64 | -0.14 |
|  |  | RF | 38.46 | 0.66 | -0.03 |
|  |  | XGB | 53.85 | 0.59 | 0.12 |
|  |  | SVR | 61.54 | 0.60 | -0.07 |
| Cluster 2 | 11 | GAM | 72.73 | 0.39 | -0.06 |
|  |  | KNN | 72.73 | 0.35 | -0.19 |
|  |  | RF | 72.73 | 0.33 | 0.12 |
|  |  | XGB | 72.73 | 0.30 | 0.40 |
|  |  | SVR | 72.73 | 0.33 | -0.14 |
| Combined Cluster Model | 24 | SVR + XGB | 66.67 | 0.46 | 0.07 |

1. **Conclusion**

In conclusion for the mindfulness dataset, our approach of clustering and training individual models on each cluster proved more effective in predicting user ratings compared to the non-clustered model. Additionally, by combining predictions from the best-performing model within each cluster, we achieved better prediction performance, further validating the benefits of our clustering approach.

**Validation of Model using Elderly Data**

1. **K Means Clustering**

The Elderly dataset, provided by Portenhauser et al. [2], contains of 55 apps with available user ratings. This dataset was divided into 70% training (n=40) and 30% testing (n=15) subsets. The elbow plot analysis [Figure 2] determined that two clusters provide the optimal segmentation.


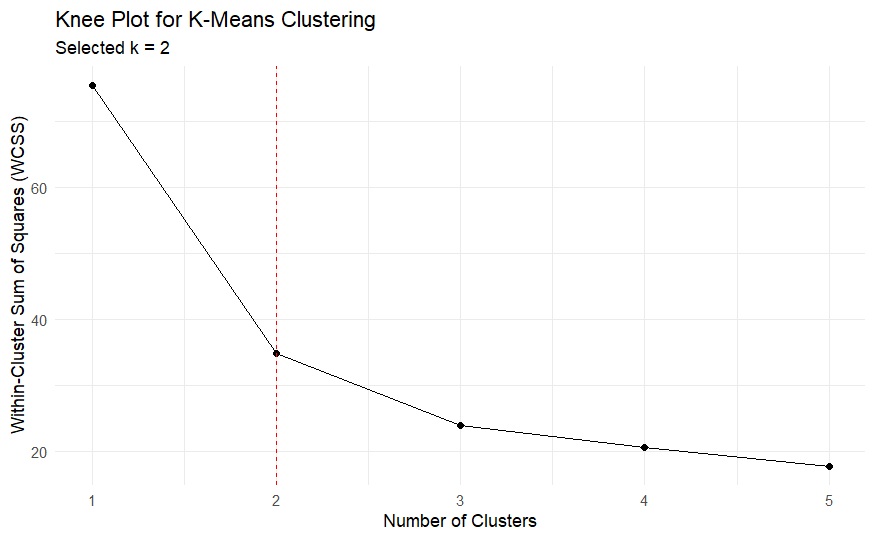


**Figure 2:** Knee Plot for K-Means Clustering showing the Within-Cluster Sum of Squares (WCSS) against the number of clusters for Elderly Data

1. **Data Distribution**

From the summary statistics table [Table 3], we see that in the unclustered dataset, user ratings and MARS scores indicate a balanced emphasis across dimensions. However, post-clustering, distinct patterns become apparent. Cluster 1 features apps with higher scores across all MARS dimensions, suggesting they closely meet user expectations for quality and offer a more comprehensive experience. In contrast, Cluster 2 displays lower scores in each dimension, representing a group of simpler, functionality-focused apps that may appeal to users seeking essential features over a rich, multifaceted experience.

**Table 3:** Summary Statistics for MARS Dimensions and User Ratings Across Unclustered and Clustered Data for Elderly Data

| **Cluster** | **Training Data Sample Size (n)** | **Dimension** | **Mean** | **Median** | **25th Percentile** | **75th Percentile** | **Range** | **Min** | **Max** |
| --- | --- | --- | --- | --- | --- | --- | --- | --- | --- |
| **Without Clustering** | 40 | **Engagement** | 3.58 | 3.60 | 3.18 | 4.20 | 3.10 | 1.70 | 4.80 |
|  |  | **Functionality** | 4.07 | 4.12 | 3.75 | 4.41 | 2.00 | 3.00 | 5.00 |
|  |  | **Aesthetics** | 3.75 | 3.75 | 3.33 | 4.17 | 3.00 | 2.00 | 5.00 |
|  |  | **Information** | 3.06 | 2.96 | 2.40 | 3.72 | 2.50 | 1.83 | 4.33 |
|  |  | **User Rating** | 4.21 | 4.35 | 4.00 | 4.53 | 3.00 | 2.00 | 5.00 |
| **1** | 15 | **Engagement** | 4.23 | 4.20 | 4.05 | 4.60 | 2.00 | 2.80 | 4.80 |
|  |  | **Functionality** | 4.48 | 4.50 | 4.25 | 4.62 | 1.00 | 4.00 | 5.00 |
|  |  | **Aesthetics** | 4.42 | 4.33 | 4.09 | 4.83 | 1.33 | 3.67 | 5.00 |
|  |  | **Information** | 3.85 | 4.00 | 3.69 | 4.21 | 1.33 | 3.00 | 4.33 |
|  |  | **User Ratings** | 4.26 | 4.50 | 4.10 | 4.60 | 3.00 | 2.00 | 5.00 |
| **2** | 25 | **Engagement** | 3.18 | 3.40 | 2.70 | 3.60 | 2.50 | 1.70 | 4.20 |
|  |  | **Functionality** | 3.82 | 3.88 | 3.50 | 4.12 | 1.75 | 3.00 | 4.75 |
|  |  | **Aesthetics** | 3.34 | 3.33 | 3.17 | 3.67 | 2.00 | 2.00 | 4.00 |
|  |  | **Information** | 2.59 | 2.50 | 2.33 | 2.90 | 1.75 | 1.83 | 3.58 |
|  |  | **User Ratings** | 4.17 | 4.20 | 4.00 | 4.50 | 2.30 | 2.70 | 5.00 |

1. **Predictions**

In the unclustered dataset, Random Forest (RF) performed best with an accuracy of 73.34% and an MAE of 0.53, though it had a slightly negative R-squared of -0.08 [Table 4].

Within the clusters, results became more defined. For Cluster 1, though small in sample size (n=2), saw KNN and SVR achieve 100% accuracy. KNN had the lowest MAE at 0.16, though R-squared remained negative, likely due to the very limited data points. Despite the limited generalizability, KNN’s accuracy and MAE suggest that it effectively captures user ratings for this group.

For Cluster 2, with a larger sample size (n=13), performance was more consistent. Both GAM and KNN achieved a correct prediction rate of 69.23%, with MAE values of 0.57 and 0.53, respectively. KNN, however, demonstrated a slightly better R-squared value of 0.03, suggesting it provided a more reliable fit for this cluster’s feature set.

The combined cluster model, applying KNN for both clusters, achieved an overall accuracy of 73.34%, with an improved MAE of 0.48 and a positive R-squared of 0.12, compared to the best unclustered model (Random Forest), which had an MAE of 0.53 and a negative R-squared of -0.08.

**Table 4:** Model Performance Metrics with and without clustering for Elderly data

| **Cluster** | **Testing Data Sample Size (n)** | **Model** | **Correct Predictions Percentage** | **MAE** | **R Squared** |
| --- | --- | --- | --- | --- | --- |
| Without Clustering | 15 | GAM | 60.00 | 0.54 | -0.10 |
|  |  | KNN | 66.67 | 0.51 | 0.07 |
|  |  | RF | 73.34 | 0.53 | -0.08 |
|  |  | XGB | 66.67 | 0.54 | -0.06 |
|  |  | SVR | 66.67 | 0.50 | -0.02 |
| Cluster 1 | 2 | GAM | 50.00 | 0.43 | -8.43 |
|  |  | KNN | 100.00 | 0.16 | -0.81 |
|  |  | RF | 50.00 | 0.53 | -18.87 |
|  |  | XGB | 50.00 | 0.51 | -12.16 |
|  |  | SVR | 100.00 | 0.27 | -3.30 |
| Cluster 2 | 13 | GAM | 69.23 | 0.57 | -0.09 |
|  |  | KNN | 69.23 | 0.53 | 0.03 |
|  |  | RF | 69.23 | 0.59 | -0.20 |
|  |  | XGB | 53.85 | 0.62 | -0.13 |
|  |  | SVR | 61.54 | 0.54 | -0.14 |
| Combined Cluster Model | 15 | KNN + KNN | 73.34 | 0.48 | 0.12 |

1. **Conclusion**

In conclusion, for Elderly apps, the clustering approach combined with individual model training for each cluster effectively reduces errors and enhances model consistency.

**References:**

1. Mani M, Kavanagh DJ, Hides L, Stoyanov SR. Review and Evaluation of Mindfulness-Based iPhone Apps. JMIR Mhealth Uhealth. 2015 Aug 19;3(3):e82. doi: 10.2196/mhealth.4328. PMID: 26290327; PMCID: PMC4705029.
2. Portenhauser AA, Terhorst Y, Schultchen D, Sander LB, Denkinger MD, Stach M, Waldherr N, Dallmeier D, Baumeister H, Messner EM. Mobile Apps for Older Adults: Systematic Search and Evaluation Within Online Stores. JMIR Aging. 2021 Feb 19;4(1):e23313. doi: 10.2196/23313. PMID: 33605884; PMCID: PMC8081158.
